# Effect of β-caryophyllene on Sperm Cryopreservation

**DOI:** 10.5935/1518-0557.20240060

**Published:** 2024

**Authors:** Emanuela Ribeiro Moura, Marlon de Araújo Castelo Branco, Yndyra Nayan Teixeira Carvalho Castelo Branco, José Adalmir Torres de Souza, Isolda Márcia Rocha do Nascimento, Marcimar Silva Sousa, Nathyelle Maria Sousa de Oliveira, Wanderson Gabriel Gomes de Melo, Dayseanny de Oliveira Bezerra, Marina Rebeca Soares Carneiro de Sousa, Daniela Kunkel, Camila Ernanda Sousa de Carvalho, Maria Christina Sanches Muratori, Antônio de Sousa Júnior, Amilton Paulo Raposo Costa

**Affiliations:** 1 Post-Graduate Program in Tropical Zootechnics/Federal University of Piaui, Teresina, Piauí, Brazil; 2 University Mauricio de Nassau, Teresina, Piauí, Brazil; 3 Post-Graduate Program in Technologies Applied to Animals of Regional Interest/Federal University of Piaui, Teresina, Piauí, Brazil; 4 Department of Veterinary Clinic and Surgery/Federal University of Piauí, Teresina, Piauí, Brazil; 5 Technical College of Teresina/Federal University of Piauí, Teresina, Piauí, Brazil; 6 Faculty of Veterinary Medicine, State University of Ceará, Fortaleza, Ceará, Brazil

**Keywords:** andrology, sheep, antioxidant, reproduction

## Abstract

**Objective:**

This study aimed to assess the impact of β-caryophyllene (BC) supplementation in the extender on the post-cryopreservation quality of semen from Dorper rams.

**Methods:**

Six Dorper rams were utilized for semen collection over 16 weeks, with BC concentrations determined via the MTT test. Animals were divided into a control group and three treatment groups receiving BC at concentrations of 1.0mM, 2.0mM, and 3.0mM in the Trisegg yolk diluent. Semen was cryopreserved and stored in liquid nitrogen for at least 15 days. After thawing, *in vitro* assessments including CASA, acrosomal integrity, plasma membrane integrity, mitochondrial membrane potential, and thermo-resistance tests were conducted. Additionally, the TBARS assay was performed to evaluate oxidative stress.

**Results:**

While BC supplementation did not significantly affect sperm motility, it notably improved mitochondrial potential and mitigated oxidative stress in cryopreserved ram semen.

**Conclusions:**

Incorporating β-caryophyllene into the extender exhibited beneficial effects on the quality of Dorper ram semen post-cryopreservation, enhancing mitochondrial functionality and reducing oxidative stress.

## INTRODUCTION

The use of cryopreserved semen from rams with superior genetics in reproductive biotechniques is one of the efficient biotechniques to promote the gain in productivity (Elsayed *et al.,* 2019; Jha *et al.,* 2019). However, this procedure provides low fertility in ewes when compared to the use of fresh semen because ovine sperm is extremely susceptible to low temperatures. This susceptibility has been attributed to the high concentrations of polyunsaturated fatty acids in the plasma membrane of sheep spermatozoa that make the cells sensitive, in the presence of reactive oxygen species (ROS) as well as to cold shock (Allai *et al.,* 2018; Amini *et al.,* 2019). Therefore, the efficiency of ram semen cryopreservation should be improved by preventing oxidative stress.

When transitioning from physiological temperature to freezing temperature, semen undergoes substantial stress on the spermatozoa’s plasma membrane, resulting in rearrangement and destabilization of cellular components, along with an influx of calcium (Padilha *et al.,* 2012). Therefore, cryopreservation subjects spermatozoa to a series of physical and chemical insults - cryoinjuries, such as the attack of free radicals, which diminishes sperm viability and fertility. They are particularly vulnerable to lipid peroxidation due to the abundance of polyunsaturated fatty acids in their plasma membrane (Mata-Campuzano *et al.,* 2015).

It is noteworthy that a low level of ROS produced as a result of oxidative metabolism is necessary to perform sperm functions such as capacitation, acrosomal reaction, hyperactivation and sperm-ovocyte fusion. Therefore, a balance must be maintained between the production and consumption of these ROS, as imbalance can lead to the onset of high rates of lipid peroxidation due to excess amounts of ROS (Gushiken *et al.,* 2022).

Post-thaw sperm viability still faces obstacles, such as excess ROS production, despite scientific and technological advances. The main cause of decreased viability due to imbalance in ROS levels are changes in ejaculate osmolarity and changes in sperm conformation during freezing and thawing procedures, which lead to decreased post-thaw fertility rates (Batissaco *et al.,* 2020).

The positive advances obtained with reproductive biotechniques can be achieved through improvements in semen cryopreservation diluents. In general, a suitable freezing extender needs, among other requirements, a system to neutralize the toxic ROS products produced by spermatozoa (Bittencourt *et al.,* 2013). Thus, it is assumed that the addition of a potent antioxidant can contribute to improve the reproductive performance of sheep by improving the viability of sperm cells after the cryopreservation process.

β-Caryophyllene (BC), a potent antioxidant, is a phytocannabinoid, abundantly found in spices such as pepper, clover, cinnamon, and oregano. BC has been shown to exert organoprotective effects against the deleterious effects of drugs, xenobiotics, or other chemical toxicants on the liver, kidney, pancreas, intestine, and brain (Al-Taee *et al.,* 2019). Various biological activities have been attributed to this natural product, such as anti-inflammatory, antibiotic, antioxidant, anticancer, and local anesthetic (Pant *et al.,* 2014). We aimed to evaluate the effect of β-caryophyllene, an antioxidant, added to the diluent on the quality of semen from Dorper breed sheep after cryopreservation.

## MATERIAL AND METHODS

### Test Substance β-Caryophyllene

The β-caryophyllene >80% purity, was obtained commercially from the company Sigma-Aldrich (Saint Louis, Missouri, USA). From a standard Tris diluent (composed of 12.11 g Tris; 6.8 g citric acid; 2.5 g fructose; 2.5 g lactose; 1 mL gentamicin, 44 mg/mL; 68 mL distilled water, 32 mL glycerol), Tris-egg yolk was prepared (composed of 60% distilled water; 20% egg yolk and 20% standard Tris diluent, osmolarity ~350 mOsm/kg and pH 6.8).

**Figure 1:**
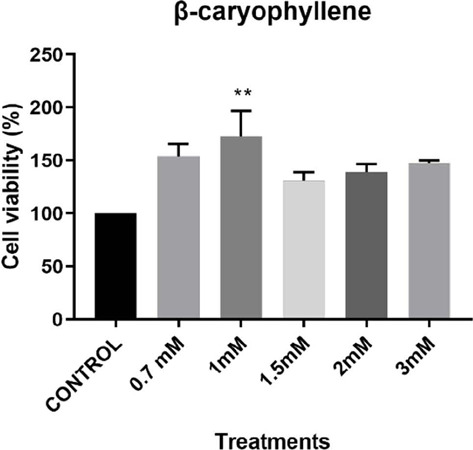
Cell viability of post-thawed ovine sperm at concentrations 0.7; 1.0; 1.5; 2.0 and 3.0mM of β-caryophyllene (BC). Values are expressed as mean±standard deviation (SD). (**)p<0.01.

In determining non-toxic concentrations of β-caryophyllene for ovine sperm, a cell viability test was conducted using concentrations of 0.7, 1.0, 1.5, 2.0, and 3.0mM (Machado *et al.,* 2020). Subsequently, based on the results of the cell viability test (MTT test), presented in Figure 1, three concentrations of BC were selected for further experimentation. Three experimental groups were formed with the following concentrations of β-caryophyllene: 1.0mM; 2.0mM and 3.0mM, added to the Tris-egg yolk diluent (Gushiken *et al.,* 2022). The Tris-egg yolk diluent, without test substance, was considered as the control group.

### Animals

Six sheep of the Dorper breed, 3 to 5 years old, from the from the Technical College of Teresina, Piauí, Brazil (Latitude:-5.047851720557213, Longitude: -42.78303633983549), of tropical Aw climate with dry season (Köppen-Geiger climate classification), and with body condition score of 3 to 4, on a scale of 1 to 5. All animals have a proven fertility history and were evaluated for general health, reproductive organ integrity and sperm quality. The animals were fed with concentrate containing 22% crude protein [3/4 corn meal + 1/4 soybean meal] and green forage, with 70% elephant grass *(Pennisetum purpureum* Schum). Mineralization was incorporated into the feed, and water was provided *ad libitum.*

### Semen collection and initial evaluation

Semen was collected once a week for eight weeks during the rainy season and another eight weeks during the dry season, totaling 96 ejaculates. The collections were made with the aid of a female in estrus, an artificial vagina and using a 15 mL graduated test tube, sterile and properly protected with aluminum foil to prevent exposure of semen to light. The test tubes with the six ejaculates were placed in a water bath at 37°C and separately evaluated for color, appearance, volume (mL), turbulence (0-5), total motility (%) and sperm vitality (1-5) under a phase contrast microscope (Olympus optical Co., Ltd., Tokyo, Japan). Sperm concentration was obtained in a Neubauer chamber, at a dilution of 1:400, in distilled water. Only ejaculates with turbulence ≥ 3; total motility ≥ 80%; vitality ≥ 3; sperm concentration ≥ 3.5 X 10^9^ sperm/mL and sperm pathologies ≤ 20% were used in this study. When approved, the samples from the six ejaculates were mixed to form a *pool,* aiming to increase the semen volume and eliminate the individual variability of the animals. After *pool* formation, it was divided in four aliquots, which were kept at 37°C in a water bath before the experimental procedures. The semen samples were collected in the rainy and non-rainy season in order to minimize the environmental effect.

### Cryopreservation of semen

The diluted semen was packed in 0.25 ml straws (50 X 10^6^ viable spermatozoa per straw) and frozen in TK 3000® machine (TK Tecnology in Freezing Ltda, Uberaba, Brazil), at the freezing curve −0,25°C/min, from 25°C to 5°C and −20°C/min, from 5°C to −120°C and, after reaching −120°C, the straws were immersed in liquid nitrogen (−196°C) and stored in a cylinder with liquid nitrogen. The equilibration time at 5°C was at least 30 minutes. After at least 15 days of storage, the semen samples were thawed in a 37°C water bath for 30 seconds and evaluated for cell viability, plasma membrane integrity, acrosome integrity, mitochondrial function, post thaw kinetics and thermo-resistance test (TTR). In addition, the thiobarbituric acid reactive substances (TBARS) test was performed to evaluate the lipid peroxidation reaction.

### Cell viability test (MTT Test)

Given the need to verify which concentrations are non-toxic for sheep spermatozoa, the cell viability test was performed using β-caryophyllene at concentrations; 0.7; 1.0; 1.5; 2.0 and 3.0mM (Ghorbani-Anarkooli *et al.,* 2019). Cell viability was assessed using the MTT test (MTT tetrazolium salt - [3-(4,5-dimethylthiazol-2-yl)-2,5-di-phenyltetrazolium bromide]), for which semen was thawed in a 37°C water bath for 30 seconds. 20µL of semen was transferred to Elisa plate containing 96 wells and 20µL of MTT solution was added to the wells containing the samples and as well as to the negative control wells. The plate was kept in an incubator with 5% CO_2_ at a temperature of 37°C for four hours. After this period, 70µL of 10% SDS (Sodium dodecyl (laurel) sulfate solution) was added and the plate was again kept in the oven overnight until reading was performed using a spectrophotometer at a wavelength of 570 nm (Souza, 2012). With regards to the results of this test, as demonstrated in Figure 1, the three concentrations of BC that were used in the other tests were selected.

### Analysis of plasma membrane integrity

To evaluate plasma membrane integrity, the double staining method was used with carboxyfluorescein diacetate (DCF; Sigma-Aldrich®, St. Louis, MO, USA) and propidium iodide (PI; Sigma-Aldrich®, St. Louis, MO, USA), modified by (Coleto *et al.,* 2002), in which 50-µL aliquots of post-thawed semen were diluted in 150µL of TRIS solution (composed of 7.210g Tris, 4.048g citric acid, 2.976g fructose and 200 mL distilled water) containing 5µL of DCF (0.46mg/mL in DMSO) and 20µL of IP (0.5mg/mL in PBS) and incubated for 10 minutes at 38°C. A total of 200 spermatozoa were evaluated under an epifluorescence microscope (Olympus optical Co., Ltd., Tokyo, Japan) at 400x magnification using DBP 580-630nm emission filter and DBP 485/20nm excitation filter. Spermatozoa were classified as having a intact membrane when stained green and a damaged membrane when stained red.

### Analysis of acrosomal integrity

To assess acrosome integrity, the fluorescein isothiocyanate dye conjugated to *Peanut agglutinin* (FITC-PNA; Sigma-Aldrich®, St Louis, MO, USA) was used according to the technique described by Roth *et al.* (1998), in which a 20µL aliquot of FITC-PNA stock solution (1mg/mL) was thawed and added to 480µL of phosphate buffered solution (PBS Sigma-Aldrich®, St Louis, MO, USA) to obtain the final concentration of 100µg/mL. Aliquots (20µL) of this solution were placed on smears of slides containing spermatozoa, which were incubated for 20 minutes in a humid chamber at 4°C in the absence of light. After incubation, the slides were rinsed twice in chilled PBS (4°C) and placed for drying in the absence of light. Immediately before evaluation, 5µL of UCD mounting medium (4.5mL glycerol, 0.5mL PBS, 5mg sodium azide, and 5mg p-phenylenediamine) was placed on the slide and covered with a coverslip. 200 spermatozoa per slide were evaluated at 1000x magnification under immersion oil in an epifluorescence microscope (Olympus optical Co., Ltd., Tokyo, Japan), using LP 515nm emission filter and BP 450-490nm for excitation. Spermatozoa were classified as having an intact acrosome when the acrosomal region was stained with green fluorescence, or as having a reacted acrosome when there was a green fluorescent band in the equatorial region of the sperm head or no green fluorescence in the entire head region.

### Mitochondrial membrane potential analysis

Mitochondrial function was determined by using a lipophilic cationic fluorochrome JC-1 (Cossarizza *et al.,* 1993). Therefore, aliquots of 50µL of post-thawed semen were diluted in 150µL of Tris containing 5µL of JC-1 (0.15mM in DMSO) and incubated for 10 minutes at 38°C. A total of 200 spermatozoa were evaluated under an epifluorescence microscope (Olympus optical Co., Ltd., Tokyo, Japan) at 1000x magnification under immersion oil, using LP 515nm emission filter and BP 450-490nm for excitation. Cells stained in orange were classified with high mitochondrial membrane potential, and those stained in green were classified with low membrane potential.

### Evaluation of sperm kinetics using integrated optical visual system

Sperm kinetics were evaluated using a computer-assisted sperm analysis system (Computer-assisted Sperm Analysis-CASA). The CASA consisted of a phase-contrast optical microscopy system (Nikon™ H5505, Eclipse 50i, Japan), with stroboscopic illumination, and a hot stage at 37°C, a video camera (Basler Vision Tecnologie™ A312FC, Ahrensburg, Germany), and a computer with Class™ sperm analyzer software (Microptics, SL, version 3.2.0, Barcelona, Spain). Sperm kinetic variables were evaluated after washing the samples in Tris medium (v/v) subsequently incubated in a 37°C water bath for 5 minutes. The variables evaluated were: progressive motility (MOP- µm/s), curvilinear velocity (VCL - µm/s), straight line velocity (VSL - µm/s), average path velocity (VAP- µm/s), linearity (LIN - %), straightness (STR-%), lateral head shift (ALH - µm), Wobble oscillation index (WOB - %), cross beat frequency (BCF-Hz) and hyperactivity, for each sperm analyzed.

### Thermoresistance Test (TTR)

The heat resistance test was performed according to Vianna *et al.* (2009), which consisted in evaluating the longevity of spermatozoa from thawed semen samples, incubated in a 37°C water bath for a period of 3 hours. The thawed samples were conditioned in 1.5 mL microtubes and incubated at 37°C, subsequently, they were evaluated for total motility (TM - %) and sperm vitality (1-5) by means of phase contrast microscopy (Olympus optical Co., Ltda., Tokyo, Japan) with attached hotplate, at 400x magnification, at 0, 60, 120 and 180 minutes post thawing according to Brazilian College of Animal Reproduction (CBRA, 2013).

### Thiobarbituric Acid Reactive Substances (TBARS) levels, malondialdehyde (MDA) concentrations

MDA concentrations were determined by thiobarbituric acid reactive substance production (TBARS) according to the method described by (Ohkawa *et al.,* 1979) with adaptations. Accordingly, 100µL of post-thawed semen was added to 175µL of 20% acetic acid (pH 3.5) and 300µL of 0.5% thiobarbituric acid. The mixture was then incubated in a water bath for 45 minutes at 100°C and subsequently cooled in an ice bath for 15 minutes. After this procedure, 25µL of 8.1% sodium dodecyl sulfate (SDS) was added to the mixture and centrifuged for 15 minutes at 12,000rpm at 25°C. 200µL of the supernatant was transferred to 96-well plate where absorbance was read at wavelength of 532 nm. A calibration analytical curve was prepared using MDA as standard at concentrations of 1.0, 5.0, 10.0, 25.0 and 50.0nmol/mL. The samples were analyzed in duplicate, and the results expressed in nmol of MDA per mL of sample.

### Statistical Analysis

Cell viability test was performed in triplicate and TBARS production in duplicate. The data were analyzed by ANOVA and the means were compared using the Tukey’s test (5% probability). Analyses were performed using Graph Pad Prism version 8 software (Graph Pad Software, California, USA).

### Ethics in animal experimentation

All experimental procedures were submitted for approval by the Ethics Committee on Animal Use (CEUA) of the Federal University of Piauí (UFPI), approval number 405/17 and certified.

## RESULTS

**Figure 2:**
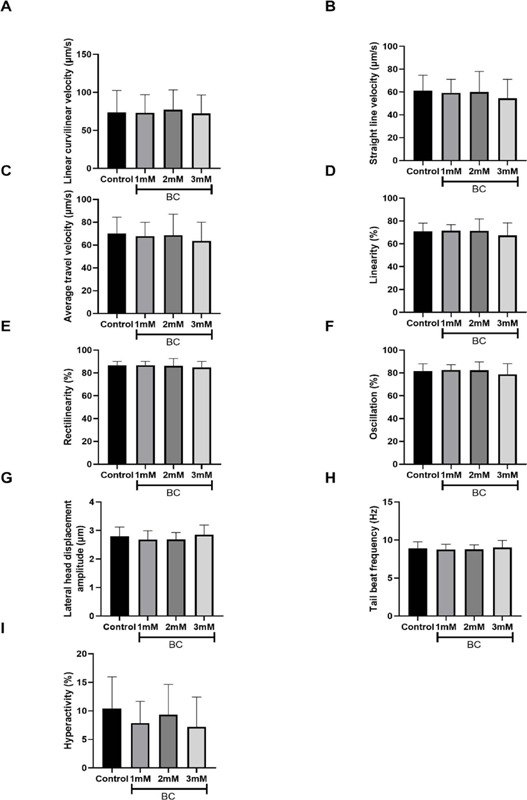
Kinetic parameters of post-thawed ovine sperm at concentrations 1.0; 2.0 and 3.0mM of β-caryophyllene (BC). Values are expressed as mean±standard deviation (SD). a) Linear curvilinear velocity (µm/s); b) Straightline velocity (µm/s); c) Average travel velocity (µm/s); d) Linearity (%); e) Rectilinearity (%); f) Oscillation (%); g) Lateral head displacement amplitude (µm); h) Tail beat frequency (Hz); and i) Hyperactivity (%). n = 16.

The result of the kinetics of cryopreserved ovine spermatozoa post-thaw at 1.0, 2.0 and 3.0mM of BC showed no statistically significant difference compared to the control group (Table 1 and Figure 2). Specifically, the control group exhibited a TM of 47.5%±4.5, with NPM and PM values of 18.7%±1.6 and 28.8%±3.5, respectively. Similarly, the treatment groups with BC concentrations of 1.0mM, 2.0mM, and 3.0mM displayed comparable motility parameters.

**Table 1:** Post-thaw kinetics of cryopreserved ovine spermatozoa at the concentrations 1.0; 2.0 and 3.0mM of β-caryophyllene (BC). Total Motility (TM); Non progressive Motility (NPM); and Progressive Motility (PM).

Treatments	TM	NPM	PM
**Control**	47.5±4.5	18.7±1.6	28.8±3.5
**BC 1.0mM**	39.8±3.8	17.2±1.4	24.1±2.6
**BC 2.0mM**	45.5±4.4	17.9±2.2	27.6±3.4
**BC 3.0mM**	41.4±4.6	18.4±2.0	23.1±3.4

Note: Results expressed as mean and standard error of mean, n = 16.

Cell viability assay, as depicted in Figure 1, revealed that the control group and the groups treated with 0.7, 1.5, 2, and 3mM of BC exhibited close to 100% cell viability. However, notably, at 1mM of BC, cell viability exceeded 150%, demonstrating statistical significance (*p*<0.01).

The plasma membrane integrity (PM), mitochondrial potential (MIT), and acrosome integrity (AC) results are presented in Table 2. The control group exhibited a PM percentage of 39.8%±3.2. Conversely, the PM percentages in the BC-treated groups were slightly higher: 45.7%±4.2 for 1.0mM BC, 43.3%±4.6 for 2.0mM BC, and 33.3%±4.7 for 3.0mM BC. Regarding MIT, the control group had a percentage of 45.4%±2.0, whereas the BC-treated groups showed varied percentages: 54.4%±1.5 for 1.0mM BC, 44.0%±4.2 for 2.0mM BC, and 43.2%±2.9 for 3.0mM BC. As for AC, the control group displayed a percentage of 58.3%±2.8, while the BC-treated groups showed differences: 67.3%±4.1 for 1.0mM BC, 53.2%±3.5 for 2.0mM BC, and 56.6%±4.5 for 3.0mM BC. Notably, BC supplementation, particularly at the concentration of 1.0mM, significantly influenced MIT, indicating an enhancement in this parameter with statistical significance (*p*<0.05).

**Table 2:** Plasma membrane integrity (PM), mitochondrial potential (MIT) and acrosomal integrity (AC) of post-thawed ovine spermatozoa at 1.0, 2.0 and 3.0mM concentrations of β-caryophyllene (BC).

Treatments	PM %	MIT %	AC %
**Control**	39.8±3.2	45.4±2.0	58.3±2.8
**BC 1.0mM**	45.7±4.2	54.4±1.5*	67.3±4.1
**BC 2.0mM**	43.3±4.6	44.0±4.2	53.2±3.5
**BC 3.0mM**	33.3±4.7	43.2±2.9	56.6±4.5

Note: Results expressed as mean and standard error of mean, n = 10, (*) *p*<0.05.

The evaluation of sperm kinetics using an integrated optical visual system revealed that the kinetic parameters measured showed no statistically significant difference in post-thawed ovine sperm at any concentrations of BC (Figure 2) (*p*>0.05).

The thermoresistance test parameters results include total motility (TM) and vitality (VG) at various time points (0, 60, 120, and 180 minutes) during exposure to elevated temperatures (Table 3). At the initial time point (0 minutes), there were no significant differences observed in TM and VG between the control group and the groups treated with BC. However, as the exposure time increased, TM gradually decreased in all groups, with no significant differences noted among the treatment groups and the control (*p*>0.05). Similarly, VG exhibited a slight decline over time across all groups, without significant differences observed among the treatment groups compared to the control (*p*>0.05).

**Table 3:** Total motility (TM) and vitality (VG) of post-thawed ovine spermatozoa at concentrations; 1.0, 2.0 and 3.0mM of β-caryophyllene (BC) submitted to the thermo-resistance test.

Parameters	Time (minutes)	Control	BC 1.0mM	BC 2.0mM	BC 3.0mM
**TM (%)**	0	34±1.6	41.0±2.8	40.0±2.6	35.0±2.2
**VG (1-5)**	0	3.0±0	3±0.1	3.1±0.1	2.9±0.1
**TM (%)**	60	35±1.7	39±2.8	36.0±2.2	35.0±1.7
**VG (1-5)**	60	3.0±0	3.2±0.1	3.2±0.1	3.0±0
**TM (%)**	120	26.0±2.2	31±2.3	32.0±2.5	26.0±2.7
**VG (1-5)**	120	2.9±0.1	2.9±0.1	2.8±0.2	2.8±0.1
**TM (%)**	180	26±2.2	31.0±2.3	32.0±2.5	26±2.7
**VG (1-5)**	180	2.5±0.2	2.8±0.2	2.8±0.1	2.5±0.2

Note: Results expressed as mean and standard error of mean, n = 10.

Regarding the levels of thiobarbituric acid reactive substances (Table 4), the results indicate that the addition of BC at concentrations of 2.0 and 3.0mM led to a significant reduction in MDA concentration compared to the control group. Specifically, the MDA concentration in semen treated with 2.0mM BC was 28.5±1.4nmol/L, while in the 3.0mM BC group, it was 28±2.7nmol/L. These values were significantly lower than the MDA concentration in the control group (45.6±4.3nmol/L). However, at a concentration of 1.0mM BC, the reduction in MDA concentration was not statistically significant compared to the control group. The MDA concentration in the 1.0mM BC group was 34±2.0nmol/L.

**Table 4:** Levels of thiobarbituric acid reactive substances (malondialdehyde -MDA) in post- thawed ovine semen at concentrations of 1.0, 2.0 and 3.0mM of β-caryophyllene (BC).

Treatments	MDA concentration (nmol/L)
**Control**	45.6±4.3
**BC 1.0mM**	34.0±2.0
**BC 2.0mM**	28.5±1.4*
**BC 3.0mM**	28.0±2.7

Note: Results expressed as mean and standard error of mean, n = 10, (*) *p*<0.05.

## DISCUSSION

Sperm kinetics is an indicator of sperm quality. The motility dynamics offered by the CASA system and traditional semen evaluations provide valuable information about semen quality before and after freezing (Batissaco *et al.,* 2020). However, the results observed in this study showed no improvement in sperm motility with addition of BC to the diluent. In contrast to the findings of Espinosa-Ahedo *et al.* (2022), which demonstrated beta-caryophyllene’s ability to alleviate sperm quality and quantity due to cadmium-induced damage. Furthermore, it corroborates the findings of Bastaki *et al.* (2020), which found no influence of beta-caryophyllene or betacaryophyllene epoxide on sperm parameters.

The analysis of plasma membrane integrity, acrosomal integrity and mitochondrial potential parameters are important because they are closely linked to semen quality, as the structural integrity of spermatozoa is necessary to maintain fertilization ability (Batissaco *et al.* , 2020). However, at the concentration of 1.0mM it promoted a higher percentage of mitochondrial matrix integrity, when compared to the control group. Thus, the addition of BC promoted an improvement in mitochondrial potential. The use of this antioxidant may be associated with a possible protection of the mitochondrial membrane, which results in a higher rate of oxidative phosphorylation and higher metabolic activity. This have a direct impact on cell motility, since mitochondria are responsible for transforming and making energy available for cell movement (Corandin, 2013).

However, the addition of BC did not promote significant effects on cell motility. Studies have shown the importance of mitochondria for sperm functionality, as the main source of ATP in cell homeostasis and motility.

Several studies demonstrate the antioxidant, antimu-tagenic, and cytotoxicity-reducing effects of beta-caryophyllene (BC) in various tissues (Di Sotto *et al.,* 2010; Viveros-Paredes *et al.,* 2017; Di Giacomo *et al.,* 2018; Wang *et al.,* 2018). The lack of correlation between the observed improvement in mitochondrial parameters and the absence of improvements in sperm kinetics can be attributed to the complexity of mechanisms of action and the different responses of cells and tissues to its effects. While BC effects on mitochondrial parameters may be more direct and immediate, influencing mitochondrial matrix integrity and membrane potential, improvements in sperm kinetics may depend on a more complex interaction of factors, such as the regulation of membrane ion channels or the synthesis of proteins related to motility (Pereira *et al.*, 2017).

The concentration of 2.0mM of BC was able to decrease the production of ROS, by reducing lipid peroxidation, but this fact did not contribute to an improvement in sperm motility. It is noteworthy that the lower the concentration of MDA, the lower its production in the medium and, consequently, the lower the occurrence of lipid peroxidation. MDA is one of the secondary products formed from the oxidation of lipids promoted by ROS. MDA is considered a biomarker of lipid peroxidation, i.e. oxidative stress (Gaschler & Stockwell, 2017). The correlation between lipid peroxidation and sperm motility was demonstrated by (Najafi *et al.,* 2014; Mehdipour *et al.* , 2017). It can be inferred that other factors may contribute to sperm motility, besides peroxidative stress reduction alone was insufficient in improving the motility of these cells.

It was concluded that although β-Caryophyllene did not increase sperm motility, it improved mitochondrial potential and attenuated oxidative stress in ram semen after cryopreservation.

## References

[r1] Allai L, Benmoula A, Marciane da Silva M, Nasser B, El Amiri B (2018). Supplementation of ram semen extender to improve seminal quality and
fertility rate. Anim Reprod Sci.

[r2] Al-Taee H, Azimullah S, Meeran MFN, Alaraj Almheiri MK, Al Jasmi RA, Tariq S, Ab Khan M, Adeghate E, Ojha S (2019). β-caryophyllene, a dietary phytocannabinoid attenuates oxidative
stress, inflammation, apoptosis and prevents structural alterations of the myocardium
against doxorubicin-induced acute cardiotoxicity in rats: An in vitro and in vivo
study. Eur J Pharmacol.

[r3] Amini S, Masoumi R, Rostami B, Shahir MH, Taghilou P, Arslan HO (2019). Effects of supplementation of Trisegg yolk extender with royal jelly on
chilled and frozen-thawed ram semen characteristics. Cryobiology.

[r4] Bastaki M, Api AM, Aubanel M, Bauter M, Cachet T, Demyttenaere JCR, Diop MM, Harman CL, Hayashi SM, Krammer G, Lu V, Marone PA, Mendes O, Renskers KJ, Schnabel J, Tsang SY, Taylor SV (2020). Dietary administration of β-caryophyllene and its epoxide to
Sprague-Dawley rats for 90 days. Food Chem Toxicol.

[r5] Batissaco L, Arruda RP, Alves MBR, Torres MA, Lemes KM, Prado-Filho RR, Almeida TG, de Andrade AFC, Celeghini ECC (2020). Cholesterol-loaded cyclodextrin is efficient in preserving sperm quality of
cryopreserved ram semen with low freezability. Reprod Biol.

[r6] Bittencourt RF, Oba E, Ribeiro Filho AL, Chalhoub M, Azevedo HC, Bicudo SD (2013). Advances in cryopreservation of ram semen I: extenders and
cryoprotectans. Ciênc Anim Bras.

[r7] CBRA. Colégio Brasileiro de Reprodução Animal (2013). Manual para exame andrológico e avaliação de sêmen
animal.

[r8] Coleto ZF, Guerra MMP, Batista AM (2002). Avaliação do sêmen congelado de caprinos com drogas
fluorescentes. Rev Bras Med Vet.

[r9] Corandin EM (2013). Avaliação da cisteína adicionada ao meio diluente sobre
espermatozoides ovinos mantidos fresco, refrigerado e congelado.

[r10] Cossarizza A, Baccarani-Contri M, Kalashnikova G, Franceschi C (1993). A new method for the cytofluorimetric analysis of mitochondrial membrane
potential using the J-aggregate forming lipophilic cation
5,5′,6,6′-tetrachloro-1,1′,3,3′-tetraethylbenzimidazolcarbocyanine iodide
(JC-1). Biochem Biophys Res Commun.

[r11] Di Giacomo S, Abete L, Cocchiola R, Mazzanti G, Eufemi M, Di Sotto A (2018). Caryophyllane sesquiterpenes inhibit DNA-damage by tobacco smoke in
bacterial and mammalian cells. Food Chem Toxicol.

[r12] Di Sotto A, Mazzanti G, Carbone F, Hrelia P, Maffei F (2010). Inhibition by beta-caryophyllene of ethyl methanesulfonate-induced
clastogenicity in cultured human lymphocytes. Mutat Res.

[r13] Elsayed DH, El-Shamy AA, Abdelrazek HMA, El-Badry DA (2019). Effect of genistein on semen quality, antioxidant capacity, caspase-3
expression and DNA integrity in cryopreserved ram spermatozoa. Small Rumin Res.

[r14] Espinosa-Ahedo BA, Madrigal-Bujaidar E, Sánchez-Gutiérrez M, Izquierdo-Vega JA, Morales-González JA, Madrigal-Santillán EO, Álvarez-González I (2022). Potential protective effect of beta-caryophyllene against cadmium
chloride-induced damage to the male reproductive system in mouse. Reprod Toxicol.

[r15] Gaschler MM, Stockwell BR (2017). Lipid peroxidation in cell death. Biochem Biophys Res Commun.

[r16] Ghorbani-Anarkooli M, Dabirian S, Moladoust H, Zendedel A, Bahadori MH (2019). Comparison of MTT, trypan blue, and clonogenic assay, to determine the
viability in human anaplastic thyroid cancer cell line. Tehran Univ Med Sci J.

[r17] Gushiken LFS, Beserra FP, Hussni MF, Gonzaga MT, Ribeiro VP, de Souza PF, Campos JCL, Massaro TNC, Hussni CA, Takahira RK, Marcato PD, Bastos JK, Pellizzon CH (2022). Beta-caryophyllene as an antioxidant, anti-inflammatory and
re-epithelialization activities in a rat skin wound excision model. Oxid Med Cell Longev.

[r18] Jha PK, Shahi Alam MG, Mansur AA, Naher N, Islam T, Uddin Bhuiyan M, Bari FY (2019). Cryopreservation of Bangladeshi ram semen using different diluents wand
manual freezing techniques. Cryobiology.

[r19] Machado KC, Paz MFCJ, Santos JVO, Silva FCC, Tchekalarova JD, Salehi B, Islam MT, Setzer WN, Sharifi-Rad J, Castro e Sousa JM, Cavalcante AACM (2020). Anxiety Therapeutic Interventions of β-Caryophyllene: A
Laboratory-Based Study. Nat Prod Commun.

[r20] Mata-Campuzano M, Álvarez-Rodríguez M, Álvarez M, Tamayo-Canul J, Anel L, de Paz P, Martínez-Pastor F (2015). Post-thawing quality and incubation resilience of cryopreserved ram
spermatozoa are affected by antioxidant supplementation and choice of
extender. Theriogenology.

[r21] Mehdipour M, Daghigh Kia H, Nazari M, Najafi A (2017). Effect of lecithin nanoliposome or soybean lecithin supplemented by
pomegranate extract on post-thaw flow cytometric, microscopic and oxidative parameters
in ram semen. Cryobiology.

[r22] Najafi A, Kia HD, Mohammadi H, Najafi MH, Zanganeh Z, Sharafi M, Martinez-Pastor F, Adeldust H (2014). Different concentrations of cysteamine and ergothioneine improve
microscopic and oxidative parameters in ram semen frozen with a soybean lecithin
extender. Cryobiology.

[r23] Ohkawa H, Ohishi N, Yagi K (1979). Assay for lipid peroxides in animal tissues by thiobarbituric acid
reaction. Anal Biochem.

[r24] Padilha RT, Magalhães-Padilha DM, Cavalcante MM, Almeida AP, Haag KT, Gastal MO, Nunes JF, Rodrigues AP, Figueiredo JR, Oliveira MA (2012). Effect of insulin-like growth factor-I on some quality traits and fertility
of cryopreserved ovine semen. Theriogenology.

[r25] Pant A, Mishra V, Saikia SK, Shukla V, Asthana J, Akhoon BA, Pandey R (2014). Beta-caryophyllene modulates expression of stress response genes and
mediates longevity in Caenorhabditis elegans. Exp Gerontol.

[r26] Pereira R, Sá R, Barros A, Sousa M (2017). Major regulatory mechanisms involved in sperm motility. Asian J Androl.

[r27] Roth TL, Weiss RB, Buff JL, Bush LM, Wildt DE, Bush M (1998). Heterologous in vitro fertilization and sperm capacitation in an endangered
African antelope, the scimitar-horned oryx (Oryx dammah). Biol Reprod.

[r28] Souza MAF (2012). Comparação de dois métodos para criopreservação
de sêmen ovino.

[r29] Vianna FP, Papa FO, Zahn FS, Melo CM, Dell’Aqua JA Jr. (2009). Thermoresistance sperm tests are not predictive of potential fertility for
cryopreserved bull semen. Anim Reprod Sci.

[r30] Viveros-Paredes JM, González-Castañeda RE, Gertsch J, Chaparro-Huerta V, López-Roa RI, Vázquez-Valls E, Beas-Zarate C, Camins-Espuny A, Flores-Soto ME (2017). Neuroprotective Effects of β-Caryophyllene against Dopaminergic
Neuron Injury in a Murine Model of Parkinson’s Disease Induced by MPTP. Pharmaceuticals (Basel).

[r31] Wang G, Ma W, Du J (2018). β-Caryophyllene (BCP) ameliorates MPP+ induced
cytotoxicity. Biomed Pharmacother.

